# Relationship between Physical Activity and the Metabolic, Inflammatory Axis in Pregnant Participants

**DOI:** 10.3390/ijerph182413160

**Published:** 2021-12-14

**Authors:** Adeline Bockler, Nina Ferrari, Clara Deibert, Anne Flöck, Waltraut M. Merz, Ulrich Gembruch, Christina Ehrhardt, Jörg Dötsch, Christine Joisten

**Affiliations:** 1 Department of General Internal Medicine/Cardiology, Marienhof Hospital, Rudolf-Virchow-Str. 7–9, 56073 Koblenz, Germany; 2 Cologne Center for Prevention in Childhood and Youth/Heart Center Cologne, University Hospital of Cologne, Kerpener Str. 62, 50937 Cologne, Germany; nina.ferrari@uk-koeln.de; 3 Department for Physical Activity in Public Health, Institute of Movement and Neurosciences, German Sport University Cologne, Am Sportpark Müngersdorf 6, 50933 Cologne, Germany; ehrhardt@nutrition-affairs.de (C.E.); c.joisten@dshs-koeln.de (C.J.); 4 Department of Pediatric, DRK Hospital Kirchen, Bahnhofstraße 24, 57548 Kirchen, Germany; clara_deibert@web.de; 5 Department of Obstetrics and Prenatal Medicine, University Bonn Medical School, Venusberg-Campus 1, 53127 Bonn, Germany; anne.floeck@ukb.uni-bonn.de (A.F.); waltraut.merz@ukb.uni-bonn.de (W.M.M.); ulrich.gembruch@ukb.uni-bonn.de (U.G.); 6 Department of Pediatrics and Adolescent Medicine, Faculty of Medicine, University Hospital Cologne and University of Cologne, Robert-Koch-Str. 16, 50931 Cologne, Germany; joerg.doetsch@uk-koeln.de

**Keywords:** pregnancy, myokine, adipokine, physical activity

## Abstract

Physical activity (PA) during pregnancy is beneficial for mother and child. Little is known regarding the effects of PA on specific adipokines/myokines and their impact during pregnancy. This study investigates the correlation between PA during late pregnancy, body composition, and maternal levels of leptin, IL-6, and TNF-α at delivery. In a cross-sectional study of 91 pregnant participants (mean age 33.9 ± 4.6 years) without gestational diabetes mellitus or preeclampsia, anthropometric data and blood samples were taken at delivery. PA during the third trimester was measured via the Pregnancy Physical Activity Questionnaire. Activities were ranked by intensity: sedentary (<1.5 metabolic equivalent (METs)), light (1.5–3.0 METs), moderate (3.0–6.0 METs), and vigorous activity (>6.0 METs). Leptin at delivery correlated positively with body composition and negatively with light PA intensity. Sedentary behaviour showed a positive correlation with IL-6 levels at delivery. Moderate activity during the last trimester, sedentary activity levels, and body composition had the greatest influence on maternal IL-6 at delivery. Completed weeks of pregnancy, moderate and light PA, and sedentary activity had the greatest influence on maternal TNF-α at delivery. PA during late pregnancy potentially affects circulating (adipo-)/myokines. Further studies are needed to examine causal relationships and the impact on maternal and new-born health.

## 1. Introduction

The benefits associated with physical activity during pregnancy for both maternal and new-born health are supported by research [1,2,3,4]. For example, regular physical activity during pregnancy has been linked to reduced risk of weight gain [5], hypertension [6], diabetes [5,6], back pain [7], mood disorders [8,9], large for gestational age [3], premature birth [3], and caesarean sections [2]. In 2020, the World Health Organization (WHO) updated physical activity and sedentary behaviour guidelines [10]. Pregnant individuals are encouraged to perform at least 150 min of moderate-intensity aerobic physical activity throughout the week. Moreover, pregnant individuals should limit the amount of time spent sedentary, and replacing sedentary behaviour with physical activity of any intensity (light, moderate, or vigorous) provides health benefits [10].

These effects may be explained by changes in metabolic and cardiovascular processes in which so-called myokines appear to play a crucial role. Similar to adipokines released by (visceral) fat mass, myokines are a variety of chemokines, peptides, and cytokines associated with adaptive processes such as glucose and/or lipid metabolism, atrophy/hypertrophy, and the cross talk between muscles and other organ systems, including fatty tissue, immune system, liver, brain, etc. [11].

In 1994, leptin was the first adipokine to be described [12]. It plays a central role in the regulation of energy balance and food intake as well as in controlling immunity and inflammation [13]. While leptin is primarily secreted by adipose tissue, it is also released by skeletal muscle [14,15]. The specific mechanisms remain unclear. It appears that leptin partitions the fatty acids away from storage in skeletal muscle and into utilisation [16]. During pregnancy, higher levels of leptin in the first and second trimesters have been associated with the development of gestational diabetes [17], whereas regular physical activity during pregnancy has been associated with a beneficial decrease of leptin levels [18].

Due to its central regulatory functions in both tissues, IL-6 is also known as adipo-myokine. In contrast to its pro-inflammatory effect as an adipocytokine, as a myokine, IL-6 may influence muscle hypertrophy, myogenesis, and fatty acid deposition in the muscle [19]. Additionally, IL-6 may act systemically, for example, by increasing exercise-induced glucose release in the liver [11]. The combination of exercise type, intensity, and duration determines the magnitude of exercise-induced increases or decreases in plasma IL-6 levels [20,21,22]. Only two studies have investigated the effects of IL-6 in pregnant individuals with regard to physical activity [23,24]. Van Poppel et al. [23] showed that higher levels of physical activity resulted in significantly higher levels of IL-6, measured at various time points during pregnancy (15, 24, and 32 weeks of pregnancy). In contrast, Acosta-Manzano et al. [24] found no significant relationship between vigorous physical activity and IL-6 at 17 weeks of gestation, after adjusting for potential confounders. Increased levels of IL-6 in physically active pregnant individuals likely represent IL-6 originating from the muscles, which appears to be anti-inflammatory, associated with increased levels of lipolysis and fatty acid metabolism and the suspension of TNF-α. In addition, these increased IL-6 levels may be related to improved insulin sensitivity [25]. However, the precise relationships between physical activity, IL-6, and inflammation remains unclear.

Similar to IL-6, TNF-α acts as an adipo-myokine. As an adipokine, TNF-α may negatively regulate many aspects of glucose and lipid metabolism [26]. Local actions of TNF-α may impact whole-body insulin sensitivity through increased free fatty acid and altered adipokine production [26]. As a myokine, TNF-α may act as an energy sensor and may inhibit myoblast differentiation among other functions [19]. Regarding physical activity, regular physical activity does not result in changes to TNF-α levels in the blood, in contrast to engaging in intense levels of physical activity (e.g., high-intensity interval training (HIT)) or long-lasting physical activity (e.g., marathons), which both lead to increased TNF-α levels [20]. To date, few studies have investigated the effect of regular physical activity on TNF-α levels during pregnancy [23,27,28]. In contrast to Van Poppel et al. [23], who only reported decreased TNF-α levels at the beginning of pregnancy, Clapp and Kiess [27] demonstrated that participants who were physically active during pregnancy showed lower TNF-α levels than participants who decreased their physical activity levels or who were inactive. Acosta-Manzano et al. [28] also found reduced TNF-α levels in the active group during pregnancy; however, when adjusting for other potential confounders, these differences became non-significant.

Little is known regarding different physical activity intensities; therefore, the present study aims to investigate the correlation between the amount and intensity of physical activity (light, moderate, vigorous, or sports exercise) and/or sedentary behaviour, body composition, and maternal levels of the adipokine leptin and the myokines IL-6 and TNF-α at delivery.

## 2. Materials and Methods

This study was performed as a cross-sectional analysis at the Department of Obstetrics and Prenatal Medicine, University Hospital Bonn (Germany), in cooperation with the German Sport University Cologne (Germany), between December 2013 and April 2014, as previously described [29,30]. A total sample of 91 participants was obtained, with all participants between 36 and 41 weeks of gestation. Exclusion criteria included participants who experienced premature birth before 36 weeks of gestation, multiple births, participants with psychiatric disorders, participants with gestational diabetes mellitus, participants with preeclampsia, participants with missing information on gestational diabetes or preeclampsia, participants with insufficient knowledge of German, and any cases in which any illness of the fetus was known in advance.

Ethical approval for this study was obtained from the University Hospital Bonn (Ethics reference number: 269/13). The study was conducted in accordance with the ethical principles of medical research on humans (Declaration of Helsinki) and the World Medical Association. All study participants signed informed consent forms affirming their voluntary participation.

### 2.1. Anthropometric Data

The following participant information was retrieved from either medical files or health insurance cards: age, height, weight (both before gestation and at present), parity, ethnicity, level of education, smoking behaviour, incidence of gestational diabetes, incidence of preeclampsia, birth procedure and complications during pregnancy.

The prenatal maternal body mass index (BMI) was calculated using the following formula: body weight (kg)/(body height (m))^2^. Weight changes during pregnancy were generated from participant medical files and were computed using the difference between the weight measured at the most recent pregnancy medical check-up during pregnancy and the weight before gestation.

In addition, on the day after delivery, two further measurements were obtained. The first was the upper arm and thigh circumference, on the right side, using a non-flexible measuring tape with an accuracy of 0.1 cm. Second, the skinfold thickness for various body parts, including the triceps, hips, front axillary line at the height of the tenth rib, and rectus femoris, was evaluated using a Harpenden Skinfold Caliper (John Bull British Indicators Ltd., Harpenden, UK) with an accuracy of 0.2 mm and constant contact pressure (10 g/mm^2^). Each body part was measured three times, and a mean value was obtained. The upper arm and thigh fat mass were estimated based on the circumference of each limb and the mean skinfold thickness, using the following formula: UFE = C × (TS/2) and TUA = C2/(4π), where UFE is the upper arm/thigh fat area estimate; C is the upper arm/thigh circumference; TS is the triceps skinfold thickness; and TUA is the total upper arm [31].

### 2.2. Selected Laboratory Parameters

A maternal venous blood sample (7.5 mL serum tube, S-Monovette, Sarstedt, Nümbrecht, Germany) was obtained, in a non-fasting state, upon admission to the delivery room. New-born blood samples (6 mL) were drawn from the placental area of the umbilical cord at the same time for each participant, immediately after the clamping of the cord and before delivery of the placenta.

The samples were stored for a maximum of 48 h at 0.4 °C, centrifuged (4000 rpm for 10 min at 4 °C in a Hettich MR20 centrifuge (Tuttlingen, Germany)), and the serum was pipetted and moved to a new tube for storage. The samples were then stored at −20 °C until evaluation.

Leptin was measured by a direct sandwich enzyme-linked immunosorbent assay (ELISA, kit from MERCK/Millipore KGaA, Darmstadt, Germany), according to the manufacturer’s instructions, using a TECAN reader (Nano Quant infinite M200 Pro, Männedorf, Switzerland). A seven-point standard curve was generated, with a minimum detection level of 0.78 ng/mL.

TNF-α and IL-6 were measured by a multiplex immunoassay (eBioscience, San Diego, CA, USA) and read using a Luminex 200 reader (Luminex, Austin, TX, USA). A seven-point standard curve was generated on each plate, with minimum detection levels of 9.1 and 6.01 pg/mL for TNF-α and IL-6, respectively (calculated with Bio-Plex Manager 6.1, Bio-Rad, Hercules, CA, USA).

### 2.3. Questionnaire

Levels of physical activity and sedentary behaviour during the third trimester were recorded retrospectively, using the semi-quantitative Pregnancy Physical Activity Questionnaire [32]. The questionnaire included 32 items which can be categorised as follows: household/caregiving (13 activities), occupational (5 activities), sports/exercise (8 activities), transportation (3 activities), and inactivity (3 activities). Responses were provided according to six options: none, <0.5 h/day, 0.5 to almost 1 h/day, 1 to almost 2 h/day, 2 to almost 3 h/day, and ≥3 h/day. The questionnaire contained two blank spaces for the addition of other activities. Each activity was assigned a metabolic equivalent (MET) for pregnant participants, which represents the metabolic rate for a given activity compared to the resting rate of the body. Each MET was multiplied by the number of minutes reported for each activity to obtain the energy consumed (MET-hours/week) for each activity [32,33]. Activities were ranked by intensity: sedentary (<1.5 METs), light (1.5–3.0 METs), moderate (3.0–6.0 METs), and vigorous activity (>6.0 METs). Sport-related activity was also determined, in min per week, based on the eight questions in the sports/exercise section of the Pregnancy Physical Activity Questionnaire. Participants completed the questionnaire themselves and had the option of completing it before delivery (e.g., at induction of labour) or one day after delivery.

### 2.4. Statistical Analysis

Data analysis was performed using IBM SPSS Statistics 25.0 software (IBM Corp., Armonk, NY, USA). Mean values and standard deviations (SD) were calculated using descriptive statistics to present anthropometric and lifestyle data. Statistical significance was defined as a *p*-Value of <0.05. All confidence intervals (CIs) were estimated at the 95% level. Correlations were used to find meaningful relationships among the data. Multiple linear regression analyses were performed to analyse individual factors impacting leptin, IL-6, and TNF-α levels at delivery.

The initial model included the following variables: maternal age, completed weeks of pregnancy, weight before pregnancy, BMI before pregnancy, weight gain during pregnancy, upper arm circumference, thigh circumference, total upper arm volume, upper arm fat percentage, sports activity (min/week), total activity (METs), sedentary activity (METs), light-intensity activity (METs), and moderate-intensity activity (METs).

The number of cases may vary in the following result section. In some cases, the evaluated blood parameters were below the detection limit and were therefore not included in the analysis. In other cases, the questionnaire was not completely filled out, or the measurement of body composition could not be performed.

## 3. Results

The mean gestational age was 38.9 ± 1.3 weeks of gestation (minimum 36 weeks of gestation; maximum 41 weeks of gestation). Maternal anthropometric and lifestyle data as well as obstetric and new-born data are displayed in Table 1 and Table 2. The mean maternal and umbilical-cord blood sample results are shown in Table 3.

Total activity during the third trimester was calculated at 267.6 ± 122.1 METs. The participants stated that they had been physically active for an average of 119.6 ± 117.0 min per week (Table 1). Thus, only 33.8% (n = 27) of the study participants met the WHO activity recommendations.

### 3.1. Leptin

For maternal body composition, positive correlations were identified between leptin at delivery and upper arm circumference (r = 0.485, *p* ≤ 0.001), between leptin at delivery and total upper arm area (r = 0.476, *p* ≤ 0.001) and between leptin at delivery and upper arm fat area estimate (r = 0.475, *p* ≤ 0.001) (see Supplementary Figure S1).

Negative correlations were identified between leptin at delivery and sports exercise performance (r = −0.264, *p* = 0.022) and between leptin at delivery and light-intensity activity (r = −0.241, *p* = 0.032) (see Supplementary Figure S2). No correlation was found between leptin at delivery and sedentary activity. Furthermore, no correlation was found between maternal and umbilical cord leptin levels.

The final model of the multiple linear regression contained the following variables: maternal age (β coefficient: −0.194, *p* = 0.041); BMI before pregnancy (β coefficient: 1.248, *p* ≤ 0.001); weight before pregnancy (β coefficient: −0.627, *p* = 0.006); weight gain during pregnancy (β coefficient: 0.268, *p* = 0.010); and sports exercise (β coefficient: −0.159, *p* = 0.090). These variables explain 51.4% of the variance (r^2^ = 0.514, Table 4).

### 3.2. IL-6

No correlation was found between IL-6 levels at delivery and body composition at delivery (upper arm circumference, total upper arm area, and upper arm fat area).

According to the physical activity data, no correlation was found between IL-6 at delivery and total activity, sports exercise, light-intensity activity, or moderate activity. However, there was a tendency towards a negative correlation between IL-6 at delivery and moderate-intensity activity (r = −0.435, *p* = 0.055) (see Supplementary Figure S3A). A positive correlation was identified between IL-6 at delivery and sedentary activity (r = 0.477, *p* = 0.033) (see Supplementary Figure S3B). A positive correlation was found between maternal and umbilical cord IL-6 levels at delivery (r = 0.651, *p* = 0.042), although it needs to be taken into account that there was a very reduced sample size (n = 10).

The final model of the multiple linear regression contained the following variables: weight before pregnancy (β coefficient: 0.860, *p* ≤ 0.001); thigh circumference (β coefficient: 0.776, *p* ≤ 0.001); total upper arm area (β coefficient: −1.419, *p* ≤ 0.001); moderate activity (β coefficient: −0.452, *p* = 0.002); and sedentary activity (β coefficient: 0.225, *p* = 0.082). These variables explain 84.0% of the variance (r^2^ = 0.840, Table 5).

### 3.3. TNF-α

No correlation was found between TNF-α levels at delivery and body composition (upper arm circumference, total upper arm area, and upper arm fat area). A positive correlation was found between TNF-α levels at delivery and moderate activity (r = 0.370, *p* = 0.013) (see Supplementary Figure S4). No correlations were found between TNF-α at delivery and other intensities of physical activity (total activity, sedentary, light or sports exercise). A negative correlation was identified between TNF-α at delivery and the maternal age (r = −0.335, *p* = 0.021). Furthermore, no correlation was found between maternal and umbilical cord TNF-α levels.

The final model of the multiple linear regression contained the following variables: completed weeks of pregnancy (β coefficient: 0.297, *p* = 0.045); light activity (β coefficient: −0.363, *p* = 0.023); sedentary activity (β coefficient: 0.293, *p* = 0.042); and moderate activity (β coefficient: 0.697, *p* ≤ 0.001). These variables explain 40.0% of the variance (r^2^ = 0.400, Table 6).

## 4. Discussion

To our knowledge, the present study is the first to examine the association between various levels of physical activity intensity during pregnancy and leptin, IL-6, and TNF-α levels at delivery and to consider which lifestyle factors may influence the levels of these adipokines/myokines.

Our study results indicate that increased physical activity was significantly associated with reduced leptin levels at delivery and moderate physical activity at the end of pregnancy was associated with a tendency towards lower maternal IL-6 concentrations. Sedentary behaviour was associated with IL-6 and TNF-α, not with leptin levels at delivery. All chosen parameters were influenced in different ways by gestational age and pregravid BMI.

Regarding leptin, our results are consistent with two different studies describing a comparable inverse relationship between physical activity and leptin levels [18,27]. As described above, Clapp et al. demonstrated a nearly linear increase in leptin levels as the pregnancy progressed; however, this progression was reduced by exercise at all time points [27]. Additionally, Ning et al. [18] demonstrated that mean leptin levels were lower in women with the highest levels of physical activity (>12.8 h/week) and energy expenditure (>70.4 MET-hours/week), compared to inactive women during early pregnancy (on average, 12–13 weeks of gestation). The positive correlation between physical activity and lower leptin levels at delivery in our study may also be explained by the beneficial influence of physical activity on body composition. Thus, leptin levels at delivery were associated with upper arm fat area and pregravid weight or BMI. High leptin levels in the first and second trimesters of pregnancy have been associated with the development of gestational diabetes [16]; thus, these findings emphasise the importance of healthy lifestyle behaviour before and during pregnancy.

More inconsistent are our findings on IL-6 and physical activity when compared to the literature [23,24]. Increased levels of physical activity during pregnancy have been associated with increased IL-6 concentrations in maternal blood [23]. Similarly, Acosta-Manzano et al. [24] suggest in their study that pregnant women with greater levels of vigorous physical activity might present higher IL-6 concentrations, although this association only tended to be significant (*p* = 0.06).

However, methodological differences between the studies and our results must be considered. Physical activity was recorded by questionnaire, from which the respective intensities were derived. In contrast, Van Poppel et al. [23] and Acosta-Manzano et al. [24] objectively measured physical activity using actigraphs/accelerometers, which allow a more precise assessment. In addition, the timing of the blood sample varied from our study (at delivery) to others (32 weeks of pregnancy [23] or 17 weeks of pregnancy [24]). However, because the authors of the previous studies did not provide precise information regarding the frequency, intensity, duration or timing between physical exercise performance and blood sample collection, comparisons are limited. Higher IL-6 levels have been observed in more active women, as noted by Van Poppel et al. [23] and in tendency by Acosta-Manzano et al. [24]; this finding might be explained by muscle-secreted IL-6. Methodologically, this is a challenge to demonstrate clearly, unless a blood sample is taken immediately before or after physical exertion. However, because the timing of blood samples obtained by the previous studies was not clearly described, only speculation is possible. In addition, these positive correlations have only been identified in studies examining high-intensity/vigorous physical activity. Moderate physical activity was reported to have no effects on IL-6 concentrations [24].

Moreover, we found a positive correlation between maternal and umbilical cord IL-6 levels at delivery. The interpretation must take into account that the data refer to a very small sample size. Therefore, any potential clinical consequences of this positive correlation between maternal and umbilical cord IL-6 levels can only be speculated.

In our study, IL-6 was associated with sedentary behaviour. Indeed, elevated levels of the adipokine IL-6 during pregnancy have been associated with various diseases in pregnancy, such as preeclampsia [34] and gestational diabetes [35]. Therefore, our results support the WHO recommendation [10] that time spent sedentary be limited. Replacing sedentary behaviour with physical activity of any intensity appears to be important.

This is partially supported by our findings regarding TNF-α, although our results are inconsistent. Higher levels of moderate physical activity in the third trimester were associated with higher TNF-α levels at delivery. In contrast, studies by Van Poppel et al. [23] and Acosta-Manzano et al. [28] reported reduced TNF-α levels in active pregnant individuals. This may be due to the time period because no further available studies have examined the effects of physical activity on TNF-α levels during late pregnancy. Rather, studies have focused on sedentary behaviour which have been positively associated with higher TNF-α levels at 32 weeks of gestation [36].

### Limitations

The present study has several limitations in addition to those already discussed. First, the present work had a cross-sectional design, which generally excludes investigation of cause–effect relationships. Although the original sample was quite large (without excluding participants with gestational diabetes and preeclampsia), including more than 100 subjects, it was not sufficiently large to generate subgroup analyses. Furthermore, participants were only recruited from the obstetric unit of the University of Bonn Medical School. Therefore, our sample may not be representative of the general population. Moreover, the increased occurrence of specific subpopulations, such as increased interest in study participation among health-conscious individuals with good fitness, cannot be ruled out. Furthermore, the number of analysable samples varied greatly. This was mainly due to the fact that blood parameters, especially IL-6 and TNF-α, were below the detection limit and therefore not included in the analysis. IL-6 and TNF-α are sensitive and respond rapidly to confounding variables such as improper sample storage, although we attempted to minimise these factors. Moreover, both IL-6 and TNF-α have short half-lives, which may have influenced our results. In addition, samples were taken only once—at the point of entrance to the delivery room when other factors, such as stress, time delay in blood collection, mode of delivery, etc., may have influenced the cytokine levels. There is some evidence in the literature that the mode of delivery may impact the level of maternal IL-6 or TNF-α at delivery [37,38]. In contrast, Kiriakopoulos et al. [39] did not find any statistically significant differences between the vaginal delivery group and the elective caesarean section group during the first stage of labour, which is comparable with our time point of blood sample collection. Moreover, a comparison between mode of delivery and blood parameters in our study showed no significant differences (data not shown).

In addition, a questionnaire was used to assess participants’ physical activity levels. Subjective measurements are widely used in epidemiological studies as they are low-cost and easy to obtain; however, their validity is limited [40]. More objective measurement methods, such as actigraphy, are clearly more precise. In future studies, a combination of objective and subjective physical activity measurements should be included.

## 5. Conclusions

In conclusion, our study shows that increased physical activity in the third trimester was associated with more favourable leptin and IL-6 levels at the time of delivery, in addition to pregravid BMI and weight gain. Meanwhile, increased sedentary activity was associated with increased IL-6 and TNF-α levels at delivery. Although we cannot draw a direct dose–response relationship with the present work, it nonetheless provides important evidence regarding the influence of physical activity on selected biomarkers and thus on the health of pregnant individuals. Therefore, our findings emphasise the WHO’s call for sufficient physical activity and limited sedentary activities during pregnancy. Additionally, further studies on the exercise secretome during pregnancy are needed to gain a better understanding of the role of myokines and adipokines, especially with regard to changes in the metabolic, inflammatory axis of the mother during pregnancy and the resulting effects on the unborn child.

## Figures and Tables

**Table 1:** Maternal anthropometric and lifestyle data.

Parameter	N	Mean ± SD/n (%)	Minimum	Maximum
Maternal Age (years)	91	33.9 ± 4.6	18.0	43.6
Weight before pregnancy (kg)	91	68.5 ± 15.9	48.0	146.0
Height (cm)	91	168.4 ± 7.1	149.0	186.0
BMI before pregnancy (kg/m^2^)	91	24.2 ± 5.3	17.1	49.4
Maternal BMI classes				
underweight (<18.5 kg/m^2^)		3 (3.3)		
normal weight (18.5–24.9 kg/m^2^)		62 (68.1)		
overweight (25–29.9 kg/m^2^)		14 (15.4)		
obese (>30 kg/m^2^)		12 (13.2)		
Weight gain during pregnancy (kg)	91	15.4 ± 5.5	4.0	30.3
Upper arm circumference (cm)	87	27.2 ± 3.7	18.0	41.0
Thigh circumference (cm)	82	50.4 ± 7.4	35.0	73.0
Total upper arm area (cm^2^)	87	59.9 ± 17.1	25.8	133.8
Upper arm fat area (cm^2^)	86	29.6 ± 13.4	7.4	106.1
Total activity 3rd trimester (METs)	84	267.6 ± 122.1	33.0	559.0
Sedentary activity 3rd trimester (METs)	84	90.9 ± 45.0	5.5	194.1
Light-intensity activity 3rd trimester (METs)	84	102.0 ± 58.5	0	259.9
Moderate-intensity activity 3rd trimester (METs)	84	74.3 ± 80.8	0	332.6
Sports exercise (min/week)	80	119.6 ± 117.0	0	465.0

Legend: BMI = Body Mass Index; MET = metabolic equivalent.

**Table 2:** Obstetric and new-born data.

Parameter	N	Mean ± SD/n (%)	Minimum	Maximum
**Mode of delivery**				
Normal vaginal delivery		44 (48.4)		
Instrumental vaginal delivery		4 (4.4)		
Elective caesarean section		34 (37.4)		
Emergency caesarean section		9 (9.9)		
**Neonatal data**				
Male sex		35 (38.5)		
Female sex		56 (61.5)		
Birthweight (g)	91	3406.2 ± 473.2	2150.0	4450.0
Length (cm)	91	51.1 ± 2.5	45.0	56.0
Head circumference (cm)	91	35.2 ± 1.3	31.0	38.0
5 min Apgar score ≥ 8		91 (100)		
5 min Apgar score ≥ 9		91 (100)		

**Table 3:** Maternal and umbilical cord blood sample results.

Blood Sample Results	N	Mean ± SD	Minimum	Maximum
Maternal leptin (ng/mL) at delivery	85	22.8 ± 17.1	1.0	83.3
Maternal IL-6 (pg/mL) at delivery	20	35.7 ± 31.1	9.7	147.2
Maternal TNF-α (pg/mL) at delivery	47	15.3 ± 4.2	4.8	25.0
Umbilical cord leptin (ng/mL)	85	9.4 ± 7.7	1.1	41.1
Umbilical cord IL-6 (pg/mL)	28	80.4 ± 180.2	5.9	926.6
Umbilical cord TNF-α (pg/mL)	68	25.8 ± 7.8	10.4	39.4

**Table 4:** Multiple linear regression: maternal leptin at delivery as an outcome variable (first and final model).

Model		Beta	*p*-Value	R^2^
1	Maternal age (year)	−0.213	0.046	0.540
	Completed weeks of pregnancy (weeks)	−0.100	0.367	
	Weight before pregnancy (kg)	−0.640	0.018	
	BMI before pregnancy (kg/m^2^)	1.064	<0.001	
	Weight gain during pregnancy (kg)	0.255	0.056	
	Upper arm circumference (cm)	0.433	0.630	
	Thigh circumference (cm)	0.079	0.662	
	Total upper arm area (cm^2^)	−0.522	0.586	
	Upper arm fat area (cm^2^)	0.243	0.244	
	Sedentary activity 3rd trimester (METs)	−0.026	0.800	
	Light-intensity activity 3rd trimester (METs)	−0.052	0.665	
	Moderate-intensity activity 3rd trimester (METs)	−0.043	0.740	
	Sports exercise (min/week)	–0.144	0.191	
11	Maternal age (year)	−0.194	0.041	0.514
	Weight before pregnancy (kg)	−0.627	0.006	
	BMI before pregnancy (kg/m^2^)	1.248	<0.001	
	Weight gain during pregnancy (kg)	0.268	0.010	
	Sports exercise (min/week)	−0.159	0.090	

Legend: BMI = Body Mass Index; MET = metabolic equivalent.

**Table 5:** Multiple linear regression: maternal IL-6 at delivery as an outcome variable (first and final model).

Model		Beta	*p*-Value	R^2^
1	Maternal age (year)	0.046	0.848	0.897
	Completed weeks of pregnancy (weeks)	−0.243	0.344	
	Weight before pregnancy (kg)	0.923	0.108	
	BMI before pregnancy (kg/m^2^)	−0.394	0.440	
	Weight gain during pregnancy (kg)	0.108	0.656	
	Upper arm circumference (cm)	0.270	0.914	
	Thigh circumference (cm)	1.057	0.032	
	Total upper arm area (cm^2^)	−2.260	0.460	
	Upper arm fat area (cm^2^)	0.695	0.367	
	Sedentary activity 3rd trimester (METs)	0.201	0.305	
	Light-intensity activity 3rd trimester (METs)	−0.235	0.459	
	Moderate-intensity activity 3rd trimester (METs)	−0.489	0.049	
	Sports exercise (min/week)	0.004	0.984	
9	Weight before pregnancy (kg)	0.860	<0.001	0.840
	Thigh circumference (cm)	0.776	<0.001	
	Total upper arm area (cm^2^)	−1.419	<0.001	
	Sedentary activity 3rd trimester (METs)	0.225	0.082	
	Moderate-intensity activity 3rd trimester (METs)	–0.452	0.002	

Legend: BMI = Body Mass Index; MET = metabolic equivalent.

**Table 6:** Multiple linear regression: maternal TNF-α at delivery as an outcome variable (first and final model).

Model		Beta	*p*-Value	R^2^
1	Maternal age (year)	−0.240	0.153	0.527
	Completed weeks of pregnancy (weeks)	0.224	0.205	
	Weight before pregnancy (kg)	0.172	0.724	
	BMI before pregnancy (kg/m^2^)	0.502	0.333	
	Weight gain during pregnancy (kg)	0.115	0.527	
	Upper arm circumference (cm)	0.896	0.525	
	Thigh circumference (cm)	−0.359	0.249	
	Total upper arm area (cm^2^)	−1.381	0.383	
	Upper arm fat area (cm^2^)	0.283	0.486	
	Sedentary activity 3rd trimester (METs)	0.233	0.159	
	Light-intensity activity 3rd trimester (METs)	−0.336	0.101	
	Moderate-intensity activity 3rd trimester (METs)	0.545	0.012	
	Sports exercise (min/week)	−0.047	0.782	
10	Completed weeks of pregnancy (weeks)	0.297	0.045	0.400
	Sedentary activity 3rd trimester (METs)	0.293	0.042	
	Light-intensity activity 3rd trimester (METs)	−0.363	0.023	
	Moderate-intensity activity 3rd trimester (METs)	0.697	<0.001	

Legend: BMI = Body Mass Index; METs = metabolic equivalent.

## Supplementary Materials

The following are available online at https://www.mdpi.com/article/10.3390/ijerph182413160/s1, Figure S1: Correlation between maternal body composition and maternal leptin level at delivery (ng/mL). Figure S2: Correlation between maternal physical activity and maternal leptin level at delivery (ng/mL). Figure S3: Correlation between maternal physical activity and maternal IL-6 level at delivery (pg/mL). Figure S4: Correlation between maternal physical activity and maternal TNF-α level at delivery (pg/mL).
