# Supplementary material for: Relationship between Physical Activity and the Metabolic, Inflammatory Axis in Pregnant Participants
Source: Int J Environ Res Public Health. 2021 Dec 14;18(24):13160. doi: 10.3390/ijerph182413160 (PMC8701987; doi:10.3390/ijerph182413160)
Supplement: Supplementary file 1 [file ijerph-18-13160-s001.zip › ijerph-1432388-supplementary.pdf]

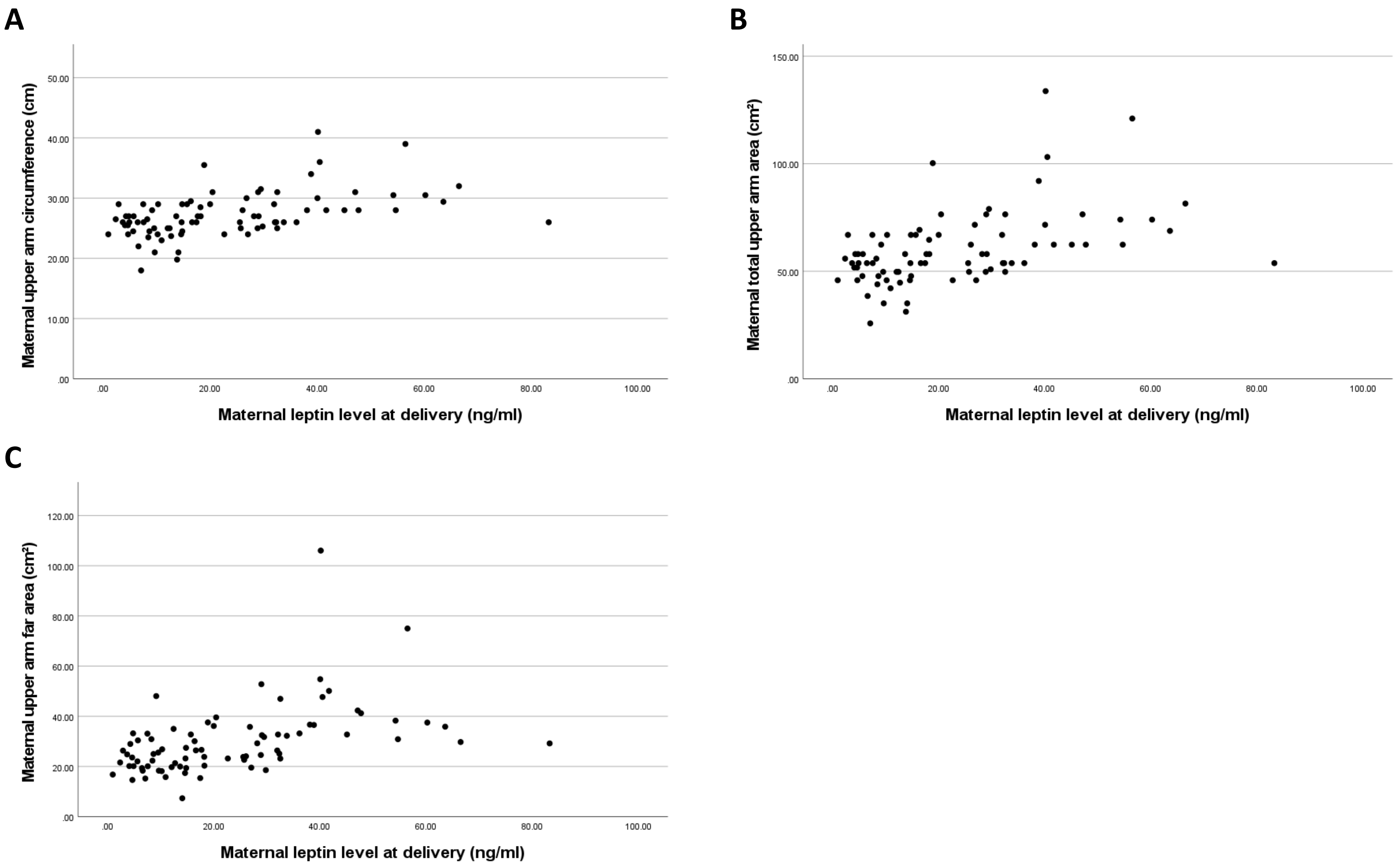

Figure S1: Correlation between maternal body composition and maternal leptin level at delivery (ng/ml); **A:** Positive correlation between maternal upper arm circumference (cm) and maternal leptin level at delivery (ng/ml); (r = 0.485, $p \leq 0.001$); **B:** Positive correlation between maternal total upper arm area (cm^2) and maternal leptin level at delivery (ng/ml); (r = 0.476, $p \leq 0.001$); **C:** Positive correlation between maternal upper arm fat area (cm^2) and maternal leptin level at delivery (ng/ml); (r = 0.475, $p \leq 0.001$)

A

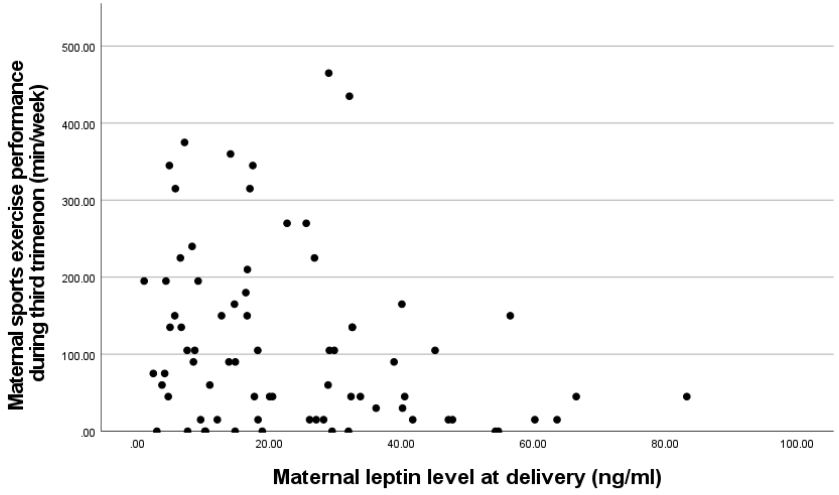

B

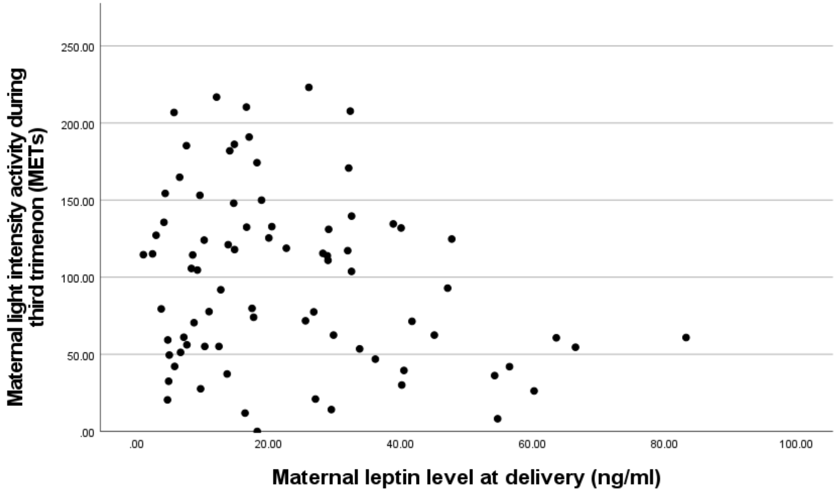

Figure S2: Correlation between maternal physical activity and maternal leptin level at delivery (ng/ml); **A:** Negative correlation between maternal sports exercise performance during the third trimenon (min/week) and maternal leptin level at delivery (ng/ml); (r = –0.264, *p* = 0.022); **B:** Negative correlation between maternal light intensity physical activity (METs) and maternal leptin level at delivery (ng/ml); (r = –0.241, *p* = 0.032)

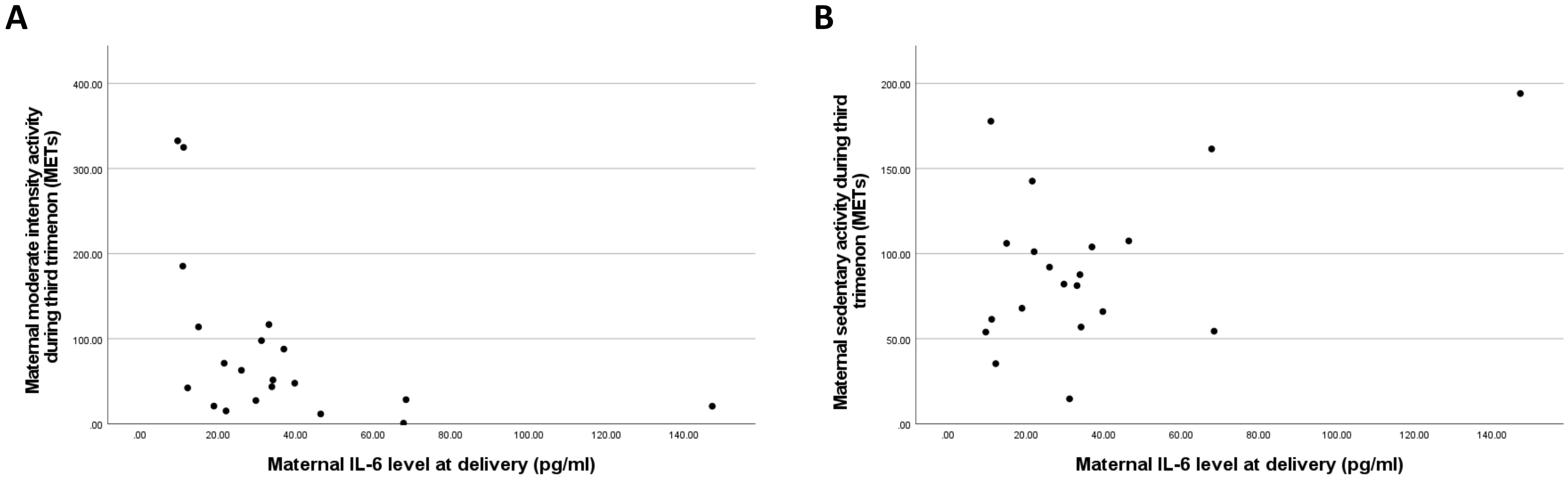

Figure S3: Correlation between maternal physical activity and maternal IL-6 level at delivery (pg/ml); **A:** Tendency towards a negative correlation between maternal moderate intensity activity during the third trimenon (METs) and maternal IL-6 level at delivery (pg/ml); (r = -0.435, *p* = 0.055); **B:** Positive correlation between maternal sedentary activity during the third trimenon (METs) and maternal IL-6 level at delivery (pg/ml); (r = 0.477, *p* = 0.033)

A

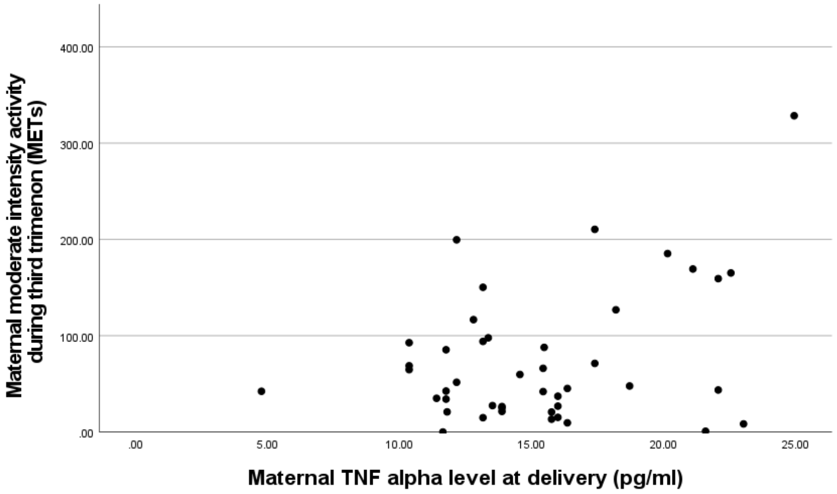

Figure S4: Correlation between maternal physical activity and maternal TNF-α levels at delivery (pg/ml); **A:** Positive correlation between maternal moderate intensity activity during the third trimenon (METs) and maternal TNF alpha level at delivery (pg/ml); (r = 0.370, *p* = 0.013)
